# CXCL12 chemokine and its receptors as major players in the interactions between immune and nervous systems

**DOI:** 10.3389/fncel.2014.00065

**Published:** 2014-03-06

**Authors:** Alice Guyon

**Affiliations:** Institut de Pharmacologie Moléculaire et Cellulaire, UMR 7275 Centre National de la Recherche Scientifique/Université Nice Sophia AntipolisValbonne, France

**Keywords:** chemokine, CXCR4, CXCR7, CXCL12/SDF-1, glutamate, GABA

## Abstract

The chemokine CXCL12/stromal cell-derived factor 1 alpha has first been described in the immune system where it functions include chemotaxis for lymphocytes and macrophages, migration of hematopoietic cells from fetal liver to bone marrow and the formation of large blood vessels. Among other chemokines, CXCL12 has recently attracted much attention in the brain as it has been shown that it can be produced not only by glial cells but also by neurons. In addition, its receptors CXCR4 and CXCR7, which are belonging to the G protein-coupled receptors family, are abundantly expressed in diverse brain area, CXCR4 being a major co-receptor for human immunodeficiency virus 1 entry. This chemokine system has been shown to play important roles in brain plasticity processes occurring during development but also in the physiology of the brain in normal and pathological conditions. For example, in neurons, CXCR4 stimulation has been shown regulate the synaptic release of glutamate and γ-aminobutyric acid (GABA). It can also act post-synaptically by activating a G protein activated inward rectifier K^+^ (GIRK), a voltage-gated K channel Kv2.1 associated to neuronal survival, and by increasing high voltage activated Ca^2+^ currents. In addition, it has been recently evidenced that there are several cross-talks between the CXCL12/CXCR4–7 system and other neurotransmitter systems in the brain (such as GABA, glutamate, opioids, and cannabinoids). Overall, this chemokine system could be one of the key players of the neuro-immune interface that participates in shaping the brain in response to changes in the environment.

## INTRODUCTION

The pathways by which nervous and immune systems interact to modulate plasticity in response to changes in the environment are still a matter of debate. It has been shown that many immune cells express receptors to neurotransmitters such as dopamine (DA), serotonin, or acetylcholine (Franco et al., 2007). Neurotransmitters released by nerve terminals in the blood or in lymphoid organs could by this way influence immune cells. Conversely, cytokines/chemokines and their receptors that were first described in the immune system have been recently found in the brain, in glial cells, and neurons themselves. Indeed, following inflammation or infection, cytokines are released in the blood. Besides their effect on the immune system, cytokines can also act in the brain to modulate our behaviors, inducing, for example, anorexia upon inflammation when produced in large amount, but cytokines/chemokines could also play a key role in the brain even in non-pathological conditions. Cytokines/chemokines can influence the brain and the behaviors through several possible pathways: modulating peripheral neurons which project to the brain through the vagus nerve, modulating the levels of hormones such as leptin which can act to the brain through the humoral pathway and acting directly in the brain, through the local production of cytokines and chemokines (Guyon et al., 2008a).

Among cytokines, chemokines are small proteins (7–14 kDa) with chemoattractant properties whose main documented role is leukocyte recruitment at inflammatory sites (Luster, 1998; Ransohoff and Tani, 1998; Glabinski and Ransohoff, 1999; Rossi and Zlotnik, 2000; Luther and Cyster, 2001; Proudfoot, 2002). At least 50 chemokines have been found to date and they have been classified as C, CC, CXC, and CXXXC according to the number and spacing of the conserved cysteine residues at the N-terminal position (Murphy et al., 2000). Phylogenic analyses showed that the large, highly redundant CXC chemokine family is a very recent phenomenon that is exclusive to higher vertebrates. Interestingly, its ancestral role might be within the central nervous system (CNS) and not within the immune system (Huising et al., 2003). Chemokines exert their biological effects through cell surface receptors that belong to the superfamily of seven-transmembrane domain G protein-coupled receptors (GPCRs).

At least 22 chemokine receptors have been characterized, which are designed following the chemokine nomenclature presented before. Most chemokines bind to several chemokine receptors and most chemokine receptors recognize several chemokines (Bacon and Harrison, 2000). Under ligand stimulation, receptor activation usually activates multiple intracellular pathways and undergoes a desensitization and internalization. Besides their role in the immune system, chemokines and their receptors seem to play an important role in the CNS, where they were first detected in immune-like competent cells (microglia and astrocytes), but were next found in neuronal cells (for review, see Cho and Miller, 2002; Banisadr et al., 2005). Local chemokine release is commonly associated to neurodegenerative and neuroinflammatory disorders such as multiple sclerosis, Alzheimer’s disease, Parkinson’s disease, and human immunodeficiency virus (HIV)-associated dementia (Streit et al., 2001; Vila et al., 2001; Lee et al., 2002; McGeer and McGeer, 2004; Cartier et al., 2005). In addition, accumulating evidence show that chemokines can modulate the electrical activity of neurons through various mechanisms (Guyon and Nahon, 2007; Rostene et al., 2007).

**FIGURE 1:**
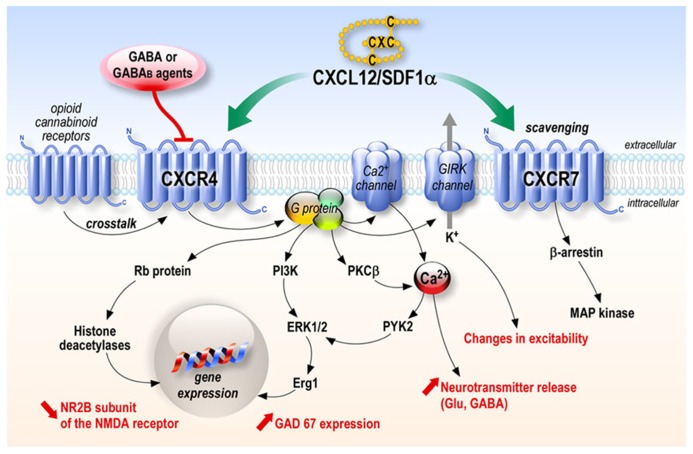
**CXCL12 chemokine signaling**. CXCL12 acts through its receptors CXCR4 and CXCR7. CXCR4 stimulation leads to the activation of numerous signaling pathways depending on the cell types while CXCR7 has mainly been shown to be involved in scavenging CXCL12, although it can activate a MAP kinase pathway through β-arrestin in several systems.

One of the most studied chemokine is the stromal cell-derived factor 1 alpha (SDF-1α) also named CXCL12. This chemokine was originally described as a secreted product of bone marrow stromal cell line (Tashiro et al., 1993). Three protein isoforms, SDF-1α, SDF-1β, and SDF-1γ, which arise from alternative mRNA splicing, have been characterized (Gleichmann et al., 2000; Pillarisetti and Gupta, 2001; Stumm et al., 2002). The SDF-1β isoform is selectively expressed by endothelial cells of cerebral microvessels and could be involved in cerebral infiltration of CXCR4-carrying leukocytes, whereas neurons synthesize SDF-1α mRNA (Stumm et al., 2002), and most studies have focused on SDF-1α. This chemokine of 67 amino acids, more recently called CXCL12, was first believed to act on a single receptor, the CXCR4. Since then, a second receptor has been found to be another target of CXCL12, namely CXCR7. Contrary to CXCR4, coupling of CXCR7 to G proteins could not be demonstrated, and CXCR7 was first believed to be mainly involved in ligand sequestration (Thelen and Thelen, 2008). However, a recent study shows that ligand binding to CXCR7 activates mitogen-activated protein (MAP) kinases through beta-arrestins (Zabel et al., 2009; Rajagopal et al., 2010; **Figure 1**).

**FIGURE 2:**
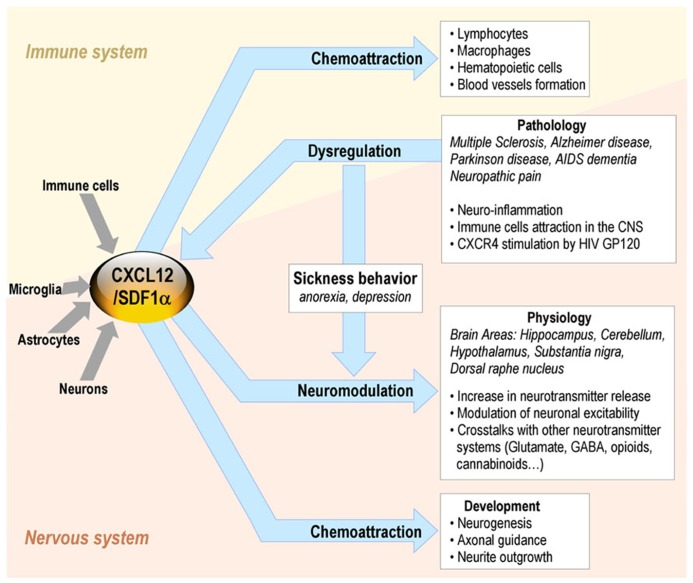
**CXCL12 roles in the brain-immune system cross-talk in non-pathological and pathological conditions**.

In the immune system, the binding of CXCL12 to CXCR4/CD184 induces intracellular signaling through several divergent pathways initiating signals related to chemotaxis, cell survival and/or proliferation, increase in intracellular calcium, and gene transcription. CXCR4 is expressed on multiple cell types including lymphocytes, hematopoietic stem cells, endothelial and epithelial cells, and cancer cells. The CXCL12/CXCR4 axis is involved in tumor progression, angiogenesis, metastasis, and survival. Besides its roles in the immune system, CXCL12 also plays a major role in the CNS (Adler and Rogers, 2005; **Figure 2**).

In a first part, we will describe the roles of this chemokine system in the brain in pathological as well as physiological conditions, and show that it is acting as a neuro-modulator. In a second part, we will show in more detail the cross-talk of the CXCL12/ CXCR4–7 system with other neurotransmitter systems in the brain, particularly γ-aminobutyric acid (GABA) and glutamate systems. Finally, we will show the role of the CXCL12/CXCR4–7 system in the immune–nervous system interaction.

## CXCL12 ACTIONS IN THE BRAIN

In the CNS, CXCL12 is an important chemokine playing a key role in *neurogenesis* (Ma et al., 1998; Zou et al., 1998; Lu et al., 2002) controlling axonal guidance and neurite outgrowth (Xiang et al., 2002; Pujol et al., 2005). For example, it is established that the future interneurons are maintained by a CXCL12/CXCR4 mediated attractive interaction in their tangential cortical routes (Stumm et al., 2003; Lopez-Bendito et al., 2008; Lysko et al., 2011). CXCL12/CXCR4 has also been shown to regulate the migration and orientation of processes in A9–A10 dopaminergic neurons (Yang et al., 2013). During development, CXCL12 also regulates the migration rate of gonadotropin-releasing hormone (GnRH) neurons (neuroendocrine cells, located in the hypothalamus, that play an essential role in mammalian reproduction), through CXCR4 and activation of a GIRK channel (Casoni et al., 2012). The migration of GnRH neurons is also indirectly influenced by CXCR7, which regulates CXCL12 availability by acting as a scavenger along the migratory path of these neurons (Memi et al., 2013).

CXCL12 also plays a major role in *neuro-inflammation* as it mediates local immune responses as well as attracting leukocytes which are believed to migrate along a concentration gradient of chemokine across the blood–brain barrier (BBB) to their target (Engelhardt and Ransohoff, 2012). This occurs for example in Alzheimer’s disease in the vicinity of the amyloid plaques that attract and/or activate local glial cells (Xia and Hyman, 1999). As the glycoprotein gp120 from the envelope of HIV-1 binds directly to CXCR4 and has direct neurotoxic effects, CXCR4 is likely to be crucial for different aspects of CNS HIV infection and the development of AIDS dementia, and CXCL12 could have *neuroprotective* effects in this context as well as in other forms of damage.

Aside from a role in CNS development and pathology, constitutive *expression* of CXCL12 and its receptor CXCR4 has been demonstrated in different cell types of the adult brain including endothelial, glial, and notably neuronal cells (Ohtani et al., 1998; Bajetto et al., 1999a; Lazarini et al., 2000; Stumm et al., 2002; Banisadr et al., 2003, 2004; Bonavia et al., 2003; Heinisch and Kirby, 2010). *In situ* hybridization and immunocytochemistry showed that CXCR4 neuronal expression was found in many different brain areas, notably cerebral cortex, globus pallidus, caudate putamen and substantia innominata (Banisadr et al., 2002), supraoptic and paraventricular hypothalamic nuclei (Banisadr et al., 2003), lateral hypothalamus (LHA; where CXCR4 is co-localized with neurons expressing the melanin-concentrating hormone (MCH; Guyon et al., 2005a), ventromedial thalamic nucleus and substantia nigra (SN; where CXCR4 is expressed on dopaminergic neurons of the pars compacta; Banisadr et al., 2002), but also on GABAergic neurons of the pars reticulata (Guyon et al., 2006), in the dorsal raphe nucleus (in serotoninergic and non-serotoninergic neurons (Heinisch and Kirby, 2010)) and in the cerebellum (where it is expressed both in Purkinje neurons and granule cells and in glial radial fibers; Ragozzino, 2002). Thus, CXCL12 and CXCR4 proteins were found co-expressed in a number of brain regions and much evidence suggest that they constitute together a functional receptor/ligand system in specific neuronal pathway.

CXCR4 stimulation by CXCL12 activates pertussis toxin (PTX)-sensitive G proteins which activate at least two distinct signaling pathways. The first pathway, involving phosphatidylinositol-3 (PI-3) kinase and extracellular signal-regulated kinase (ERK)1/2, has been described in rodent astrocytes, neuronal progenitors, and cortical neurons (Bacon and Harrison, 2000; Lazarini et al., 2000; Bajetto et al., 2001; Bonavia et al., 2003). The other pathway involves the phospholipase Cβ whose activation leads to an increase in the intracellular calcium in astrocytes, cortical neurons, and cerebellar granule cell, as well as in primate fetal neuron and microglia (Bajetto et al., 1999b; Klein et al., 1999; Zheng et al., 1999). The increase in calcium leads to the activation of proline-rich tyrosine kinase (PYK2), which may itself lead to ERK1/2 activation (Bajetto et al., 2001). CXCR4 stimulation can directly modulate ionic channels of the plasma membrane in neurons, particularly high-threshold calcium channels (Guyon et al., 2008b), and this could also result in intracellular calcium increase and PYK2 activation (Lazarini et al., 2003). Finally, in primary cultures of neurons, CXCR4 can also inhibit cAMP pathways through the Gi component of GPCRs (Liu et al., 2003).

The *neuromodulatory actions* of CXCL12 have been found in various neuronal populations (including CA1 neurons of the hippocampus, granular and Purkinje cells of the cerebellum, MCH neurons of the LHA, vasopressinergic neurons of the supraoptic and the paraventricular nucleus of the hypothalamus, dopaminergic neurons of the SN, serotoninergic and non-serotoninergic neurons of the dorsal raphe nucleus; Limatola et al., 2000; Ragozzino, 2002; Guyon et al., 2005a, b, 2008b; Callewaere et al., 2006; Heinisch and Kirby, 2010).

CXCR4 activation by its ligand can modulate neuronal activity through multiple regulatory pathways including and often combining: (i) modulation of voltage-dependent channels (sodium, potassium, and calcium; Limatola et al., 2000; Guyon et al., 2005b), (ii) activation of the GIRK channels, (iii) increase in neurotransmitter release (GABA, glutamate, DA), often via calcium-dependent mechanisms (Guyon and Nahon, 2007). From one structure to another, CXCL12 has often similar consequences on neuronal transmembrane currents, but through different mechanisms.

CXCL12 has *pre-synaptic actions*, which are similar in the different brain structures where it has been tested, increasing glutamate and/or GABA synaptic activities in LHA (Guyon et al., 2005a), hippocampus (Zheng et al., 1999), cerebellum (Limatola et al., 2000), SN (Guyon et al., 2006), and dorsal raphe nucleus (Heinisch and Kirby, 2010). However, the mechanisms of action of CXCL12 vary from one structure to the other: for example, the increase in frequency of GABA type A (GABA_ A_) post-synaptic events in response to CXCL12 occurs through an indirect mechanism involving glutamate release in the cerebellum (Limatola et al., 2000) and the serotoninergic neurons of the raphe nucleus (Heinisch and Kirby, 2010), while the effect is direct through CXCR4 of dopaminergic neurons in the SN (Guyon et al., 2006). Similarly, the glutamate release is tetrodotoxin (TTX) dependent in the LHA (Guyon et al., 2005a) and in the raphe (Heinisch and Kirby, 2010), while it is TTX independent in the SN (Guyon et al., 2006).

The target effects on the *post-synaptic* neurons also vary depending on the structure and the neuronal population. For example, the CXCL12-induced increase in GABA release in the LHA evokes a tonic GABA_ A_ current in MCH-expressing neurons (Guyon et al., 2005a), opposite to what is observed in DA neurons in which GABA type B (GABA_ B_) receptor stimulation following GABA spillover activates a GIRK current (Guyon et al., 2006). This could be due to various subunit compositions of the GABA_ A_ receptor expressed in the two neuronal populations, with different kinetics, and/or different subcellular localization of the GABA_A_/_B_/CXCR4 receptors and GIRK channels. Interestingly, in MCH neurons, CXCL12 also induced the activation of a GIRK current, but this happened directly through CXCR4 stimulation.

In the dorsal raphe nucleus, CXCR4 stimulation by CXCL12 stimulates spontaneous inhibitory post-synaptic potential (sIPSC) frequency, by a pre-synaptic mechanism on 5HT-neurons, but it acts on sIPSC amplitude by a post-synaptic mechanism in non-5HT neurons (Heinisch and Kirby, 2010). Finally, CXCR4 stimulation is able to modulate various voltage-dependent channels: Na^+^ and K^+^ channels of the action potential in MCH neurons (Guyon et al., 2005b) and high voltage activated (HVA) Ca channels, in particular of the N-type, in DA neurons of the SN (Guyon et al., 2008b) and in pre-synaptic glutamatergic terminals of the hippocampus (Zheng et al., 1999).

In conclusion, from one structure to another, CXCL12 has often similar consequences on neuronal transmembrane currents, but through different mechanisms.

Therefore, the CXCL12/CXCR4–7 system exerts neuromodulatory functions in the normal brain.

## CROSS-TALK WITH OTHER NEUROTRANSMITTER SYSTEMS

### CROSS-TALK WITH GABAergic SYSTEM

γ-Aminobutyric acid is the major inhibitory neurotransmitter in the adult nervous system but it also plays important roles in CNS development by regulating neurogenesis and synaptogenesis (LoTurco et al., 1995; Somogyi et al., 1995). In contrast to its inhibitory actions on adult neurons, GABA is capable of depolarizing neuronal progenitor cells and immature neurons (Ben-Ari, 2002; Rheims et al., 2008) and participates in formation of a primitive network-driven pattern of electrical activity called the giant depolarizing potentials (GDPs), an electrical circuit pattern critical to generate large oscillations of intracellular calcium for activity-dependent modulation of neuronal growth and synapse formation (Ben-Ari, 2002). HIV-1 gp120, which binds and stimulates CXCR4, enhances GDPs in neonatal rat hippocampus (Kasyanov et al., 2006), underlying the role played by CXCR4 in the developmental process. Moreover, the developmental function of GABA is in part regulated by GABA production, a process mediated by glutamic acid decarboxylases (GADs), the key rate-limiting enzymes for synthesis of GABA. Two GAD isoforms, GAD65 and GAD67, are expressed in the adult nervous system (Erlander et al., 1991). It has been shown that CXCL12/CXCR4 signaling, via ERKs and the transcription factor Egr1, induces expression of GAD67 in embryonic hippocampal cultured neurons, a mechanism which may promote the maturation of GABAergic neurons during development (Luo et al., 2008).

In adult brain, as previously mentioned, CXCL12, through CXCR4, is also able to modulate pre-synaptic GABA release (Limatola et al., 2000; Guyon et al., 2006; Heinisch and Kirby, 2010). GABA acts post-synaptically through its receptors. GABA_ A_ receptors are ionotropic receptors permeant to chloride. As CXCR4, GABA_ B_ receptors are GPCRs that mediate metabotropic action of GABA and are located on neurons and immune cells as well. Using diverse approaches, a novel interaction between CXCR4 and GABA/GABA_ B_ receptor agonists/antagonists has been recently reported, which was revealed to be an allosteric action of these agents on CXCR4 (Guyon et al., 2013). This result came first from the observation that GABA_ B_ antagonists and agonists and even GABA itself blocked CXCL12-elicited chemotaxis in human breast cancer cells, and that a GABA_ B_ antagonist blocked the potentiation by CXCL12 of high threshold Ca^+^^2^ channels in Rat dopaminergic neurons (Guyon et al., 2013). CXCR4 and GABA_ B_ often co-express in the same cell type (Banisadr et al., 2002), have complementary functionality and may be involved in cross-talk (Duthey et al., 2010). CXCR4 and GABA_ B_ receptor could have interacted directly through heterodimerization. Indeed, heterodimerization is known to play a role in signal transduction of other metabotropic receptors, for example, GABA_ B_ receptors interact with metabotropic glutamate receptors (Hirono et al., 2001). Following CXCL12 interaction, CXCR4 undergoes a homo-dimerization which is necessary for its functionality and signaling (Mellado et al., 2001; Toth et al., 2004). Dimerization, which is accompanied by receptor phosphorylation as well as changes in signal transduction processes (Rodriguez-Frade et al., 2001), enables the activation of the JAK/STAT (Janus kinase/signal transducers and activators of transcription ) pathway which allows the subsequent triggering of G protein-dependent signaling events (Vila-Coro et al., 1999). CXCR4 could also form heterodimers with other GPCRs, which could lead to complex responses according to the chemokines/peptides/neuromediator environment present in the extracellular medium. For example, CXCR4 has been shown to form heterodimers with CCR2 and CCR5, delta opioid receptors and CXCR7 (Percherancier et al., 2005; Pello et al., 2008; Levoye et al., 2009; Sohy et al., 2009). However, by co-expressing in the membrane of *Xenopus* oocytes GABA_ B_ receptors tagged with Td tomato (red fluorophore) and CXCR4 receptors tagged with green fluorescent protein (GFP), data obtained in total internal reflection fluorescence (TIRF) microscopy showed that CXCR4 and GABA_ B_ receptors did not co-localize in the membrane (A. Guyon, unpublished data).

**FIGURE 3:**
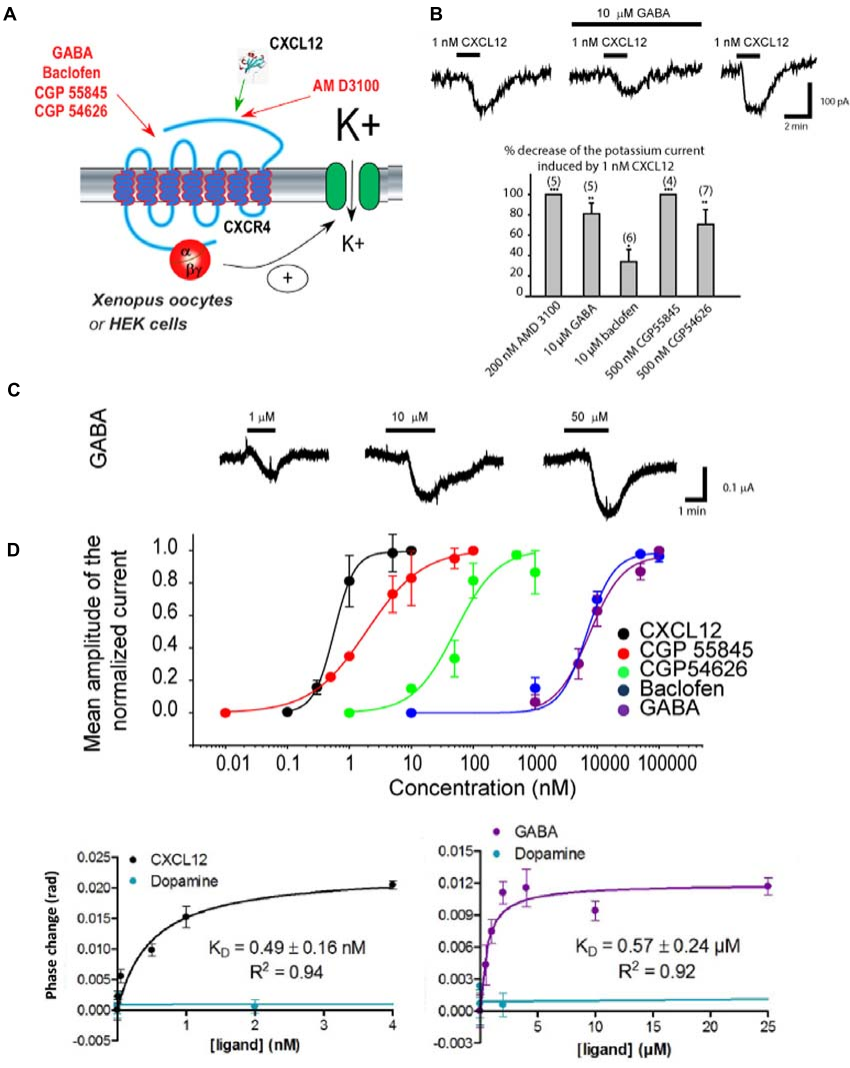
**GABA and agonists/antagonists of the GABA_B_ receptor are allosteric modulators of CXCR4**. **(A)** Scheme showing the diverse agents acting on the CXCR4 receptor that have been tested. CXCR4 has been co-expressed together with GIRK channels in *Xenopus* oocytes and human embryonic kidney (HEK293) cells. The activation of CXCR4 by the diverse agent tested was evaluated by measuring the amplitude of the GIRK current activated. **(B)** Results obtained in HEK293 cells expressing CXCR4 and reporting GIRK channel. *Top*: Traces recorded in response to the application of 1 nM CXCL12 with or without GABA. GABA inhibits partially the CXCL12 effect and this effect is reversible upon washout of GABA. *Bottom*: Histogram showing the allosteric actions of the CXCR4 antagonist AMD3100 compared to GABA and other agonists/antagonists of GABA_ B_ receptors. **(C)** Results obtained in *Xenopus* oocytes expressing CXCR4 and reporting GIRK channel. *Top*: Traces recorded in response to the application of three increasing GABA concentrations. *Bottom*: We took advantage of the agonist effect of GABA and of the GABA_ B_ agonists/antagonists in this expression system to build their concentration–response curve on CXCR4. **(D)** Back-scattering interferometry (BSI) results obtained on lipoparticles containing only CXCR4. *Left*: BSI reveals a *K*_ D_ of CXCL12 on its receptor of 0.49 ± 0.16 nM, which is coherent with the EC50 measured by our electrophysiological experiments. Dopamine, used as a negative control, does not bind to CXCR4. *Right*: BSI reveals that GABA can also directly bind to CXCR4 with a *K*_ D_ of 0.57 ± 0.24 μM, coherent with the EC50 measured in electrophysiology. Therefore, GABA can directly bind to CXCR4. Adapted from Guyon et al. (2013).

γ-Aminobutyric acid and the agonists/antagonists of GABA_ B_ receptors were recently found to act directly on CXCR4 in an allosteric manner (**Figure 3A**). Using electrophysiology in *Xenopus* oocytes and human embryonic kidney (HEK293) cells in which Rat CXCR4 and the GIRK channel were co-expressed, it could be demonstrated that GABA_ B_ antagonist and agonist modify the CXCL12-evoked activation of GIRK channels (**Figures 3B,C**; Guyon et al., 2013). By expressing CXCR4 receptors in heterologous systems lacking GABA_ B_ receptors and performing competition binding experiments it could be investigated whether GABA_ B_ ligands bind to CXCR4. FRET experiments suggested that GABA_ B_ ligands do not bind CXCR4 at the CXCL12 binding pocket suggesting allosteric modulation, in accordance with electrophysiology data (Guyon et al., 2013). Finally, back-scattering interferometry (BSI) on lipoparticles containing only the CXCR4 receptor allowed to quantify the binding affinity for the GABA_ B_ ligands (including GABA), which were in a similar range to the affinity of the ligands for GABA_ B_ receptors themselves, thus confirming that GABA and GABA_ B_ receptor ligands directly interact allosterically with the CXCR4 receptor (**Figure 3D**; Guyon et al., 2013).

As mentioned previously, CXCR4 activation by CXCL12 increases pre-synaptic neurotransmitter release and particularly GABA release in several neuronal populations (Guyon and Nahon, 2007; Bhattacharyya et al., 2008; Qu et al., 2008). If GABA can in turn block the effects of CXCL12, this could represent a negative feedback loop for pre-synaptic chemokine release. Indeed, when applying CXCL12 for several minutes, a transient increase in the frequency of sIPSCs is frequently observed, followed by a reduced activity (see Figure 3 in Guyon et al., 2006). This reduction could be due to an antagonistic effect of GABA, although desensitization of CXCR4 itself cannot be excluded. Similarly, it has been shown that elevated concentrations of CXCL12 exert opposite effect than lower concentrations on the electrical activity of some neuronal populations that receive GABA inputs (Guyon and Nahon, 2007). The antagonistic effect of GABA released pre-synaptically in response to CXCL12 could contribute to these biphasic effects. In the future, it will be of interest to search for putative effects of GABA_ B_ receptor ligands on CXCR7, the other receptor for CXCL12. In addition, the effect of GABAergic agents on CXCR4 suggests new therapeutic potentials for neurological and immune diseases.

### CROSS-TALK WITH GLUTAMATERGIC SYSTEMS

Glutamate is the main excitatory neurotransmitter in the adult brain. CXCL12/CXCR4 also plays a major role in the regulation of crucial components of glutamatergic transmission.

The chemokine CXCR4 receptor is a GPCR widely expressed on glial cells (especially astrocytes and microglia). Activation of the astrocytic CXCR4 by CXCL12 results in a long chain of intracellular and extracellular events [including the release of the pro-inflammatory cytokine tumor necrosis factor alpha (TNF-α) and prostanglandins] leading to glutamate release (Cali et al., 2008; Cali and Bezzi, 2010). Similarly, and as previously mentioned, CXCL12/CXCR4 increases glutamate release from neurons in different structures including LHA (Guyon et al., 2005a), hippocampus (Zheng et al., 1999), cerebellum (Limatola et al., 2000), SN (Guyon et al., 2006), and dorsal raphe nucleus (Heinisch and Kirby, 2010).

In addition, recent studies showed that CXCL12 protects cortical neurons from excitotoxicity by promoting the function of the gene-repressor protein Rb, which is involved in the recruitment of chromatin modifiers [such as histone deacetylases (HDACs)] to gene promoters. In neurons, Rb controls activity-dependent genes essential to neuronal plasticity and survival, such as the *N*-methyl-D-aspartic acid (NMDA) receptor’s subunit NR2B (Khan et al., 2008), the expression of which in the tetrameric ion channel largely affects calcium signaling by glutamate. CXCL12 selectively inhibits NR2B expression *in vitro* and *in vivo* altering NMDA-induced calcium responses associated with neuronal death, while promoting prosurvival pathways that depend on stimulation of synaptic receptors (Nicolai et al., 2010).

### CROSS-TALK WITH OTHER SYSTEMS

In the periaqueducal gray (PAG), a pretreatment with CXCL12 desensitized the analgesic effects of *opioids* (Szabo et al., 2002; Chen et al., 2007) and a heterologous desensitization mechanism at the GPCR level involving CXCR4 has been suggested (Chen et al., 2007; Heinisch et al., 2011). Moreover, intermediate opioid peptides (pro-enkephalin A-derived peptides secreted by adrenal subcapsular cell hyperplasia) have been shown to be potent activators of CXCR7 (Ikeda et al., 2013).

In the same line, the analgesic activity of the *cannabinoid* agonist WIN 55,212-2 in the brain can be overcome in situations in which there are elevated levels of CXCL12 in the brain (Benamar et al., 2008). There could be a functional interaction between chemokine and cannabinoid systems in the brain as the thermoregulatory action of the cannabinoid agonist WIN 55,212-2 in the preoptic anterior hypothalamus can be antagonized by elevated levels of CXCL12 (Benamar et al., 2009). This could explain why conditions associated with elevated level of chemokines may result in reduction of cannabinoid functions, as is the case with most neuroinflammatory diseases (such as multiple sclerosis and HIV encephalitis).

## ROLE IN THE IMMUNE–NERVOUS SYSTEM INTERACTION

As in the context of the immune system, where low levels of CXCL12 (100 ng/ml) are attractive, whereas higher levels (1 mg/ml) are repulsive for T cells (Zlatopolskiy and Laurence, 2001), CXCL12 often appears to have opposite effects on neuronal function depending on the concentration. For example, in DA neurons, at low concentrations, it acts as a neuromodulator by potentiating K^+^-induced DA secretion and HVA calcium currents, whereas at higher concentration, it decreases DA release and HVA calcium currents. This can be paralleled to what happens in MCH neurons of the LHA, where CXCL12 also exerts opposite effects on the action potential discharge depending on the concentration (Guyon et al., 2005a). Several putative mechanisms for these opposite effects, which are not mutually exclusive, are reviewed in Guyon and Nahon (2007).

CXCL12 can act in the CNS as a classical neuromediator under normal conditions and modulate the activity of several neuro-endocrine networks. Low concentrations of CXCL12 exert a tonic inhibition on MCH neurons, which are known to have a hyperpolarized membrane potential under basal conditions, as compared to orexin neurons of the LHA which are in intrinsic state of membrane depolarization and lack CXCR4 expression (Eggermann et al., 2003). In addition, the CXCR4 antagonist AMD 3100 has its own effects when applied alone which suggests that a tonic activation of CXCR4 occurs, at least in slices preparations, and that low levels of CXCL12 are secreted under basal conditions (although the slice preparation could also be considered as an inflammatory state).

However, pathological state (altered immune response or inflammation) leads to abnormal concentrations of chemokines and/or their presence at unusual sites can be found, due to local production by glial and/or endothelial cells and/or diffusion and transportation through the vascular circulation. This enhanced production of chemokines could interfere with their normal functions, affect neuronal and neuro-endocrine activity and modify the functioning of the brain, leading to pathological behaviors and/or neurotoxicity.

Following inflammation, cytokines are released in the blood and can reach the brain, as the BBB permeability is increased. Cytokine stimulation leads to higher levels of CXCL12 and other chemokines by activation of glial or endothelial cells which release chemokines (Meucci et al., 1998; Ohtani et al., 1998; Lee et al., 2002). The chemokines released can reach neurons expressing CXCR4, bind their neuronal receptors and induce a change in their excitability that could induce an adaptive answer to the inflammation, leading to the “sickness behavior,” characterized by depression, anorexia, and fatigue (Reichenberg et al., 2001; Dantzer and Kelley, 2007). Given the abundance of chemokines and their receptors in the CNS, it is not surprising that perturbations of cytokines/chemokines levels during inflammation are causing multiple perturbations in the brain functions and behaviors. The effects of CXCL12 on dorsal raphe neurons could underlie depressive symptoms frequently observed with inflammation (Maes, 1999), as dysfunction of the serotoninergic systems is implicated in depression. Similarly, the effects of CXCL12 on MCH neurons which are part of the circuits controlling feeding behavior and metabolism (Nahon, 2006) could explain the anorexia. These symptoms of sickness behavior are usually reversible when inflammation stops. However, a prolonged inflammation, producing higher levels of CXCL12 could even lead to neurotoxicity and to neuro-degenerescence (Gerashchenko and Shiromani, 2004).

## GENERAL CONCLUSION

CXCL12 have recently attracted much attention because this chemokine seems to play an important role as intermediate in the brain between cytokines and neurons in the cascade linking inflammation to adaptive behavioral changes. Convergent data suggest that CXCL12 could also act in the CNS as a classical neuromediator under normal conditions and could modulate the activity of several neuroendocrine networks. However, during a pathological state (altered immune response or inflammation), elevated concentrations of CXCL12 and/or its presence at unusual sites, due to its local production by glial and/or endothelial cells and/or its diffusion and transportation through the vascular circulation could affect neuronal and neuroendocrine activities and modify the functioning of the brain, leading to pathological behaviors and/or neurotoxicity. In addition, recent evidence show that there are numerous cross-talks between CXCL12/CXCR4–7 systems and other neurotransmitter systems of the CNS, illustrating new pathways by which the CNS and immune system can interact.

## Conflict of Interest Statement

The author declares that the research was conducted in the absence of any commercial or financial relationships that could be construed as a potential conflict of interest.
